# COVID-19 and beyond: development of a comprehensive telemedical diagnostic framework

**DOI:** 10.1007/s11548-021-02424-y

**Published:** 2021-06-06

**Authors:** Jonas Fuchtmann, Roman Krumpholz, Maximilian Berlet, Daniel Ostler, Hubertus Feussner, Sami Haddadin, Dirk Wilhelm

**Affiliations:** 1 Minimally Invasive Interdisciplinary Therapeutical Intervention, Klinikum rechts der Isar, Technical University Munich, Munich, Germany; 2 Chair of Robotics and System Intelligence, Munich School of Robotics and Machine Intelligence, Technical University Munich, Munich, Germany; 3 Department of Surgery, Klinikum rechts der Isar, Technical University Munich, Munich, Germany

**Keywords:** Telemedicine, COVID-19, Tele-examination, Teleoperated robotic system, Physical examination, Telediagnostics

## Abstract

****Purpose**:**

During the COVID-19 pandemic, a threatening bottleneck of medical staff arose due to a shortage of trained caregivers, who became infected while working with infectious patients. While telemedicine is rapidly evolving in the fields of teleconsultation and telesurgery, proper telediagnostic systems are not yet available, although the demand for contactless patient–doctor interaction is increasing.

****Methods**:**

In this project, the current limitations were addressed by developing a comprehensive telediagnostic system. Therefore, medical examinations have been assessed in collaboration with medical experts. Subsequently, a framework was developed, satisfying the relevant constraints of medical-, technical-, and hygienic- aspects in order to transform in-person examinations into a contactless procedure. Diagnostic steps were classified into three groups: assisted procedures carried out by the patient, teleoperated examination methods, and adoptions of conventional methods.

****Results**:**

The Telemedical Diagnostic Framework was implemented, resulting in a functional proof of concept, where potentially infectious patients could undergo a full medical examination. The system comprises, e.g., a naso-pharyngeal swab, an inspection of the oral cavity, auscultation, percussion, and palpation, based on robotic end-effectors. The physician is thereby connected using a newly developed user-interface and a lead robot, with force feedback control, that enables precise movements with the follower robot on the patient’s side.

****Conclusion**:**

Our concept proves the feasibility of a fully telediagnostic system, that consolidates available technology and new developments to an efficient solution enabling safe patient-doctor interaction. Besides infectious situations, this solution can also be applied to remote areas.

## Introduction

Since the coronavirus pandemic has overcome the world, important new implications for telemedicine have emerged. Every day, millions of people need to be tested for coronavirus infection using throat swabs performed by medical personnel. Assuming that the risk of transmission is higher for healthcare professionals that manipulate a patient’s oral cavity or nose, this interaction is critical for health safety. Although patient handling in testing facilities can be carried out quite safely through the usage of gowns, protective eyewear, and mouthguards, contact between medical personnel and patients in an outpatient department or clinic also poses dangers. For example, patients with an asymptomatic viral contagion could infect medical staff and other patients during their non-virus-related visit to the hospital or practice. In such cases, the great potential of telediagnostic is concealed. If it were possible to physically separate medical staff from patients while allowing them to examine as naturally as possible, the risk of virus transmission would be eliminated.

In the past, telediganostic has proven itself, e.g., in the form of a low-threshold offer used to increase accessibility for those who are not able to participate in the common health care system. Thus, so-called *health kiosks* have been investigated whether they can provide healthcare access among an African American population in Pennsylvania, USA. Up to 90% of participants found the concept useful and would use the offer again for health self-management [1, 2]. Thereby, telemedicine is not a new concept. For more than 20 years, telesurgery is applied, e.g., by use of the ZEUS system, which has proven to make even transatlantic cholecystectomy possible. However, this type of surgery has not been as useful due to the high cost and widespread availability of surgeons in developed countries [3]. Although this is a great demonstration of the potential of teleoperation, few applications have found their way into clinical practice.

First of all, telediagnostic currently has more implications than teleoperation. Thus, it enables physicians to monitor patients suffering from chronic disease or even in orthopedics [4, 5].

As part of the current eHealth movement, more and more devices for remote diagnosis and monitoring are becoming available in the private sector as well. Thus, parameters such as blood pressure, heart rate, and blood oxygenation can be assessed by the patients and become available to the absent physicians. The current coronavirus pandemic requires new developments of solutions that transfer available techniques and technology to a professional level while aiming for reducing infection rates through contact avoidance. In the following, we present our concept and an examination cabin that takes this step.

## Methods

The overall project is based on today’s medical procedure for potentially infectious patients when presenting themselves at the hospital. Current examinations and diagnostic measurements for assessing a patient’s medical condition are considered as the gold standard, and thus representing requirements for a telediagnostic check-up.

To estimate the necessities and properties of a future telemedical system satisfying all requirements, a structured interview with a group of medical experts at the university hospital *Klinikum rechts der Isar of the Technical University of Munich* was conducted. Seven of the nine experts were medical doctors and two were medical assistant personnel. For the structured interview, we made use of a self-developed questionnaire. Overall, it was differed between three diagnostic categories regarding invasiveness and diagnostic yield (I-III). The first category contained only noninvasive examinations, whereas the second category already included technical equipment. The third category included diagnostic tools that interfere with bodily integrity, as blood sampling and biopsy. Thus, experts were asked about examination techniques as well as the extent of diagnostic categories necessary for creating a proper diagnostic telemedical system. Furthermore, requests regarding the future interface for physicians conducting a telemedical procedure were gathered.

**Fig. 1:**
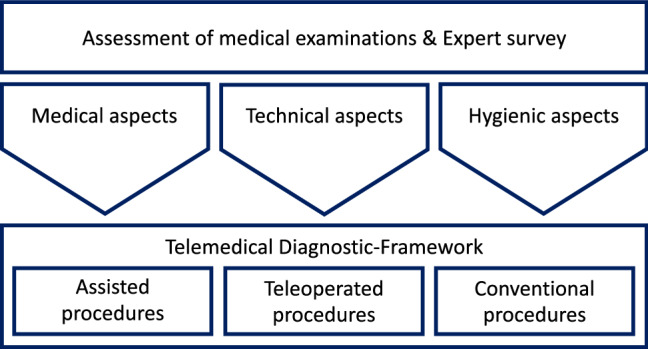
The diagram depicts the methodical approach used within in the overall project. Based on the review of standard medical examinations, three major pillars have been identified to be crucial for further project realization. Thereupon, the TDF was developed

While the outcome of the survey is listed in the result section, the further methodology is subdivided into two subsections. In Sect. 2.1, an overview of the composed key aspects for the realization of the project is given. As shown in Fig. 1, a set of three pillars was defined, namely medical-, technical-, and hygienic aspects. Definition of these pillars guided the project subsequently to the creation of the telemedical diagnostic framework (TDF), which is described in Sect. 2.2. The TDF is a composition of all necessary factors to transform a conventional examination into a telemedical procedure.

### Three pillars of telediagnostics

Based on the findings of the survey and the assessment of current examinations, three main pillars for the development of a telediagnostic system were identified.

#### Medical aspects

The medical part comprises the chain of individual examinations that a patient passes when seeing a doctor. For optimization of the process, a brief check of the vital parameters including heart rate, blood pressure, oxygen saturation of the blood, and temperature is carried out by assistant staff. Afterward in an in-depth conversation, the medical history and current condition are evaluated by the physician. Subsequently, a series of manual examinations are performed, depending on the assessments of the physician. These include, e.g., the inspection of the oral cavity, inspection of the ear, auscultation of the chest on both sides (back and front of the patient), palpation of the abdomen, or percussion along the upper body. In a final step, samples for laboratory analysis are collected including at least a swab of the pharyngeal- and nasal- cavity as well as blood samples.

#### Technical aspects

The blood pressure and heart rate are typically measured using an oscillometric blood pressure screen. To determine the oxygen saturation, a handheld device including a small finger clip is used. Both devices commonly are not connected with the central clinical information system (CIS), and registration of the findings is carried out manually.

Inspection of the ear is executed with the help of an otoscope, consisting of a light source and magnifying lenses. Consequently, the physician is adjusting the device’s position manually in order to optimize the view of the eardrum and ear channel. Check of the oral cavity does in general not require specialized tools, even though the otoscope or a wooden spatula for depressing the tongue may be used.

While palpation and percussion are accomplished without any tools, solely relying on the hands of the physician, the auscultation is only possible with the help of a stethoscope.

A conventional butterfly needle is used to draw the blood samples and a swab kit utilized for the smear.

#### Hygienic aspects

Usually, only all used devices are disinfected by wiping the surface with a disinfection agent after each and every patient. However, when dealing with potentially infectious patients, all surfaces in direct contact, including the furniture, have to be cleaned. In addition, medical staff is required to wear personal protective equipment, such as disposable gloves, gowns, eye-wear, a filtering mask, and head covering. Devices such as otoscopes provide disposable earpieces, and the entire blood sampling system is designed for single use. Regarding the contamination of air, specialized ventilation systems are usually not installed and manual airing is up to the physician.

### Telemedical diagnostic framework

To transform a medical examination into a telediagnostic procedure, the concluded findings in Sect. 2.1 had to be considered and translated into a new examination, feasible without the physical presence of the physician. Therefore, all necessary diagnostic steps were redesigned while taking the medical-, hygienic-, and technical-requirements into account. A re-classification regarding the executability of the tasks is thereby the pivotal component. Hence, three major classes were framed:

1. Assisted procedures
2. Teleoperated procedures
3. Conventional procedures

#### Assisted procedures

The first class of examination techniques was grouped as tasks, that can be carried out by the patients themselves guided by the physician, thus the name “assisted procedures.”

As the medical history of a patient is obtained by an individual series of questions, an interview of a trained medical expert is necessary. Seamless audio and video connection is thereby a prerequisite.

Although devices for measurement of body temperature and blood pressure are well-established, knowledge of handling individual devices cannot be assumed, hence requiring guidance. While most temperature measurement devices provide disposable earpieces, cuffs of blood measurement devices have to be manually disinfected. The setup time for the following patient can be shortened when using thin disposable plastic covers or multiple cuffs. Latter can be disinfected off-site. Additionally non-contact or thermal imaging solutions for measuring the patient’s temperature can be considered.

Oxygen saturation of the blood is usually measured via a noninvasive finger clip. Since this type of device is not commonly used in everyday life, instructions are needed as well. Disinfection is easily executable on-site or by swapping the finger cap as described for the blood pressure cuff. As for the otoscopy, conventional otoscopes have to be digitized using a camera to meet the needs of a telemedical application. While hygienic disposable earpieces can be used, instructions by a physician are required for placing the device at the optimal angle.

Furthermore, all above-mentioned devices require a digital interface to transmit the information to the physician.

#### Teleoperated procedures

Within the second class, diagnostic procedures are grouped, which can only be conducted by the doctor and therefore have to be performed using a teleoperated system. In this class, special handling of the diagnostic tools is necessary or the patients may not be capable of conducting the procedure on their own, e.g., auscultate their own back.

The implementation of the auscultation, percussion, and palpation has to be compatible with the wide variety of anatomical body shapes. To come up with a lean and telediagnostic setup, which can accommodate patient’s individuality, we propose the use of a robotic platform. Because of the patient’s individual anatomy, a full automation is not yet feasible. However, for diagnostics using teleoperation, force feedback is required as the robotic end-effectors are in direct contact with the patient.

Inspection of the oral cavity and the naso-pharyngeal swab presents even higher safety risks, due to the required insertion of instruments into a body orifice. Thus, fail-proof protection is indicated, limiting the patient-device interaction by design and physical constraints. Accordingly, the relevant end-effectors only need to move within a very limited range as the position of mouth and nose can be immobilized at a defined location.

#### Conventional procedures

The third class comprises tasks that still have to be executed in a conventional manner due to technical complexity or hygienic barriers. Drawing blood is an invasive procedure and therefore highest safety standards need to be applied. Moreover, finding a suitable vein and inserting the needle can even be challenging for trained medical staff. Therefore, the procedure cannot yet be automatized or teleoperated. While a conventional method of blood sampling is indicated, an implementation should eliminate the risk of contamination entirely. Therefore, the physical separation of the medical caregiver and patient has to be ensured.

#### Hygienic constrains

Besides the technical aspects, the creation of suitable hygienic concepts is important. Therefore, we interviewed experts of the hygienic department at our university hospital and created a comprehensive concept. The coronavirus by itself is not enveloped and thus is sensitive to common disinfectants containing ethanol or methanol [6]. Using ultraviolet light for disinfection has to be complemented by traditional wipe disinfection because of potential virus particles that may remain in protein-containing droplets. More importantly, since coronavirus is airborne, a proper ventilation system is required [7, 8].

## Results

First, detailed results of the expert survey are presented, followed by the realization of the Telemedical Diagnostic Framework. Here, the detailed implementation of each individual examination step is presented.

### Results of the expert survey

#### Classical examination techniques

**Fig. 2:**
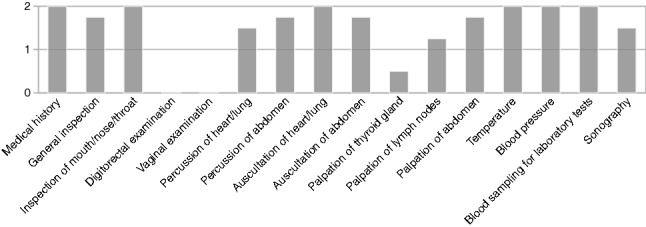
Expert opinion on key telemedicine examination modalities in the context of coronavirus pandemic, nine experts have been interviewed for their opinion on the necessity of individual examination modalities, the results have been averaged, 0 $=$ not important, 1 $=$ important, 2 $=$ mandatory

Basically, all interviewees gave priority to the medical history including the chief complaint, as well as to the classical examination modalities such as inspection of the mouth and throat as well as auscultation of the lungs. While auscultations of the lungs and abdomen were of high to very high importance, overall opinion on percussion was not consistent. Several respondents saw the importance in the age of ultrasound as rather low, but no one actually could imagine a system without the possibility of percussion of the heart, lung, and abdomen. As depicted in Fig. 2, palpation of the thyroid gland was not particularly important to the participants, while lymph nodes, e.g., on the neck, were requested to be examined.

#### Technically assisted diagnostics

Concerning technically assisted diagnostics, particular emphasis was placed on the importance of temperature and blood pressure measurement as well as blood sampling and subsequent laboratory diagnostics, as depicted in Fig. 2. The majority of respondents would like to have a sonography option but did not consider it mandatory in contrast to a controllable camera. Regarding data transfer, there was a demand for the information obtained to be incorporated seamlessly into the local clinical information system (CIS).

#### Interaction and control

Physical proximity or direct eye contact via a glass pane was requested by very few, provided that a high-quality video transmission was available. Force feedback, e.g., when placing the stethoscope on the thorax, was considered important by all medical experts.

#### Hygiene and safety

Special attention was paid to the possibility of disinfecting robotic and diagnostic equipment. The room inventory should also be easily and, if possible, automatically cleaned. As a minimum safety standard, an emergency stop button for the patient, the examiner, and further personnel involved was consistently requested.

**Fig. 3:**
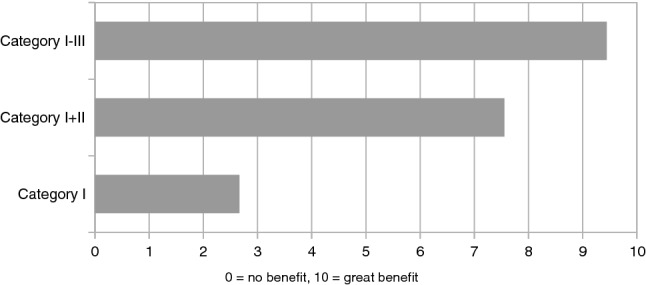
Expert opinion on the benefit from the availability of the individual diagnostic categories, 9 experts have been interviewed

#### Acceptance of a hypothetical system

**Fig. 4:**
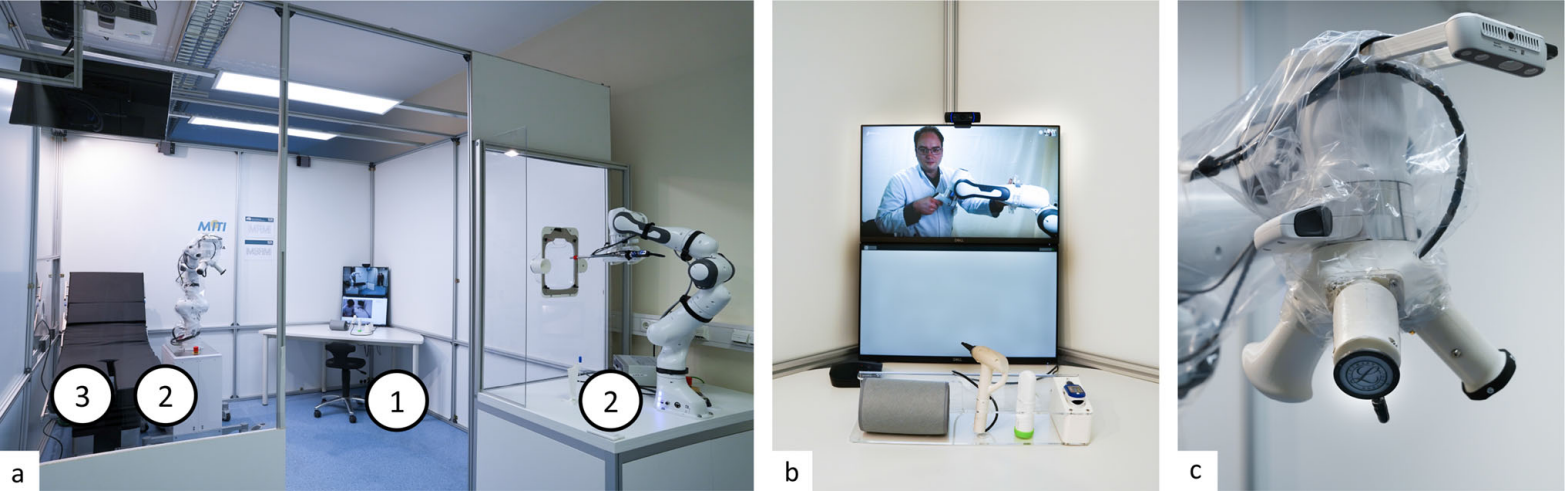
**a** Photograph of the examination cabin. White circles indicate the different examination stages. *1* Assisted examinations, *2* teleoperated examinations, *3* conventional examinations **b** detail, showing the setup of stage 1 with a video-audio stream, a wireless blood-pressure cuff, otoscope, thermometer and oximeter. **c** Detail of the robotic arm of stage 2, with the revolver like end-effector comprising an auscultation, a percussion and palpation device

Almost all respondents considered the hypothetical system in the context of a suspected infectious disease such as *COVID-19* to be useful. Almost none of the experts feared a replacement of human staff by a telemedical system. Considering the diagnostic categories, the experts agreed that the planned system would be of great benefit as soon as at least category I and II (e.g., auscultation and nasopharyngeal swabbing) would be covered. However, the greatest benefit would come from a combination of all three diagnostic categories I–III (inclusion of invasive diagnostics such as blood sampling), as shown in Fig. 3. The experts agreed that acceptance of a telediagnostic system depends heavily on how it replicates classic examination techniques. These may not be as sophisticated as modern imaging techniques, but they give the patient the feeling of the actual presence of the examining physician. So, from this point of view, it would be highly advisable to allow telemedicine practitioners to use examination methods such as auscultation, percussion, or palpation. All the more, these classical methods are crucial to get a ‘look and feel‘ of the patient’s condition, not only on a medical or technical level but also in a personal and psychological context. The consensus of the expert interviews was then that the classical examination techniques should not be replaced by such a telemedical system, rather they must be further developed and modernized. One possible solution is depicted in Fig. 4.

### Implementation of the telemedical diagnostic framework

**Fig. 5:**
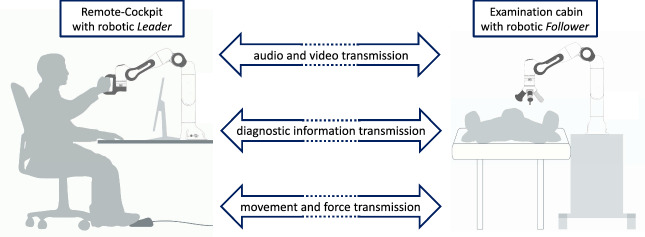
Schematic drawing of the remote cockpit with the robotic *Leader* and the examination cabin with the *Follower*. The two sites are connected via bi-directional transmission of audio and video, diagnostic information and movement and force feedback. Thereby, distance between cabin and cockpit is arbitrary, and both even do not have to be inside the same building

*Telemedical diagnostic system* The TDF and the survey resulted in the implementation of an examination cabin that can be controlled from distance via a remote cockpit. The examination cabin represents any room, e.g., in a clinic or a doctor’s practice, where the patient goes for basic diagnostics. The doctor, on the other hand, is situated in the remote cockpit, from which the patient is examined remotely. Thereby, the distance between the cabin and cockpit is arbitrary, and both are not required to be inside the same building. As indicated in Fig. 5, the medical specialist may be in a hospital, whereas the patient stays in a GP’s practice.

**Fig. 6:**
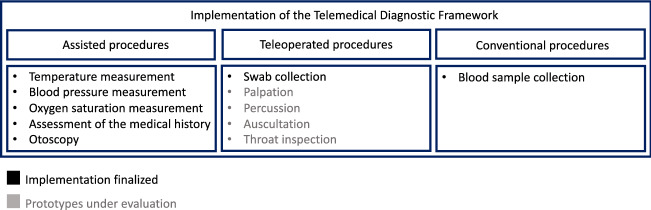
Overview, showing the current implementation of the proposed Telemedical Diagnostic Framework. All procedures have been addressed under the consideration of hygienic requirements. While multiple devices are yet ready to use for an in depth evaluation, others are at the stage of functional prototypes

*Examination cabin* The examination cabin as shown in Fig. 4 consists of three stages. All assisted examinations take place in stage 1, whereas only teleoperated examinations are performed in stage 2, while conventional examinations are carried out in stage 3. The cabin has a square basic shape and a floor area of 9 m^2^ with a ceiling height of 2.5 m. Two walls are transparent, which ensures permanent visual contact between the patient and the medical assistant. The cabin is equipped with two standard quality cameras and one controllable pan-tilt-zoom room camera with HD resolution. This enables the doctor to monitor the examination and inspect the patient. The setup comprises also two screens, which allows the patient to see the doctor. Vocal communication between physician and patient is ensured by room microphones and loudspeakers. Furthermore, the cabin has an examination couch, an automatic swap robot, and a free-standing robotic arm called the *Follower*. All implemented devices for the examination and their current status of realization are presented in Fig. 6.

*Remote-cockpit* The remote cockpit, in which the doctor is located, has a screen including a webcam and microphone. Furthermore, a robotic arm, the *Leader* is provided. This enables the audio-visual communication from the remote cockpit with the patient as well as the manipulation of the *Follower* within the cabin. The *Follower* mirrors every movement of the *Leader*. *Leader* and *Follower* are connected via a Robot Operating System Network. Another major component of the remote cockpit is the control interface of the TDF. The interface visualizes the current forces acting on the robot tip, measured vital parameters, and the examination progress. It can also be used to change and activate robotic instruments. The user interface is designed in form of a web application, to provide independence from the used operating system.

*Force feedback* The drive of the selected robotic arm is torque-controlled, which allows force determination at the tip of the robot. In combination with an additional integrated 3-DOF force sensor and force limits implemented in the software, the risk of injury to the patient can be minimized. This enables the system to provide force feedback to the physician.

*Network* On the examination cabin’s site, data of all instruments used (thermometer, otoscope, microphones, etc.) are collected by means of a real-time computer. The swab robot and the robotic arms, the *Leader* as well as the *Follower*, each have their own computing unit. At the remote cockpit, another real-time computer enables audio-visual communication. The real-time computers, robotic arms, and the swab robot communicate via TCP within a common network. Apart from the local network, the system can be used in a wide area network, provided that security and performance are ensured.

#### Assisted procedures

As depicted in Fig. 4b, Stage 1 consists of a table on which a screen, webcam, loudspeakers, and an instrument magazine are located. The instrument magazine contains a blood pressure cuff, a pulse oximeter, an optical thermometer, and an otoscope. All assisted examinations take place at this stage. The real-time computer of the cabin is used for the data transfer of all mentioned devices between the remote cockpit and stage 1.

*Medical history* The doctor starts obtaining the medical history via the audio-visual interface at stage 1. All essential medical information, as well as formal data on the patient’s social situation, is collected. The aim is to gain a first visual impression (e.g., general signs of illness, skin color, sweating) as well as to instruct the patient for the upcoming examinations. For the medical history, audio-visual communication between doctor and patient is sufficient.

*Oxygen saturation* The PO-250 finger clip (Pulox, Cologne, Germany) is used to measure the patient’s oxygen saturation. To perform the measurement, the doctor asks the patient to place the index finger of the right hand in the finger clip. An Arduino Uno reads the measurements of the pulse oximeter and transmits the data to the real-time computer, which in turn transmits the data to the remote cockpit.

*Blood pressure* To measure the blood pressure, a BPM Core wireless blood pressure cuff (Withings, Land-Issy-les-Moulineaux, France) is used. The BPM Core is a wireless upper arm cuff with an integrated display. While the display shows the results immediately after measurement, the data are also transferred to the manufacturer’s cloud via the WiFi-network of the examination cabin. A PHP script collects the data via an API and displays it on the remote-cockpit site.

*Temperature measurement* For measuring the patient’s temperature, the ear thermometer ThermoScan$^{\textregistered }$ 7 (Braun, Kronberg, Germany) is used. More convenient, contactless forehead thermometers are currently under evaluation regarding reliability and precision. To perform the measurement, the doctor instructs the patient to insert the thermometer into one of his ears. To avoid cross-contamination, disposable protective probe covers are provided, which are placed on the thermometer before each measurement. With the help of the provided cameras, the doctor controls the execution of the examination. Using the Bluetooth interface of the thermometer, the data are transmitted to the real-time computer of the cabin, from where it is forwarded to the remote cockpit.

*Otoscopy* In the last-assisted examination, a self-developed otoscope is used, which is based on an ear funnel, a magnifying lens, and a tiny camera including illumination. The camera, which is connected to the real-time computer, enables the doctor to assess the condition of the patient’s ear canals and eardrum. In addition, the doctor checks the performance of the procedure with the help of the webcam. If necessary, the physician corrects the patient with instructions. As for the temperature measurement, disposable protective probe covers are provided.

*Cleaning and processing* For an increased throughput, ten units of each medical device are provided per examination cabin. Thus, after a patient’s examination, the medical assistant only has to replace the used devices with cleaned ones. At the end of the day, all potentially contaminated equipment is sanitized by wipe disinfection.

#### Teleoperated procedures

Stage 2 provides a modified SR-NOCS swab robot (Franka Emika, Munich, Germany), an examination couch, and a Panda robotic arm (Franka Emika), the already mentioned *Follower*. The automatic swab robot is based on another Panda robot arm, which is equipped with two instruments. It enables an optical throat examination as well as a nasopharyngeal swab. The swab robot is shielded from the examination cabin by an acrylic glass pane. The *Follower*, located next to the examination couch, is used for auscultation, percussion, and palpation of the patient. For this purpose, the *Follower* is equipped with three instruments, which are stored in an instrument-revolver. Comparable to the tool magazine on CNC milling machine, the doctor can change the desired instrument by rotating the revolver by 120 degrees.

*Throat inspection and swab collection* The swab robot has an opening in the acrylic glass pane at the level of the patient’s head. A new plastic mask is attached to this opening before each patient. The mask has two protrusions, each with an opening. The plastic mask adds a physical safety barrier in case of a malfunction and prevents the instruments from being pushed too far forward, thus injuring the patient. For the sampling, the patient first puts their nose on the smaller one of the two protrusions, marked by a nose symbol. As soon as the patient has taken the correct position, the doctor gives the order to take the swab. For this purpose, the Panda arm of the machine inserts a cotton swab into the patient’s nose up to a resistance-induced position and then performs a circular movement. The cotton swab is then packed into a plastic tube provided for this purpose.

For the following pharyngeal inspection, the Panda changes to a reusable rigid Hopkins II telescope (Karl Storz, Tuttlingen, Germany). The patient now moves to the mouth protuberance of the plastic mask. The Panda arm guides the endoscope through the plastic mask into the patient’s throat. Via the control interface of the remote cockpit, the position of the endoscope can be changed in a defined area by the physician.

*Auscultation* A specially developed adapter is used for the auscultation of the patient. The adapter, shown in Fig. 4c, is based on a modified Classic III stethoscope (3M Littmann, Saint Paul, United States). The modification foresees the integration of a heavy-duty microphone of the 4660 series (DPA Microphones, Alleroed, Denmark) into the stethoscope head, with which the heart and lung sounds can be picked up and digitized. With the help of a flexible suspension of the stethoscope based on silicone, it is possible to minimize mechanical interference on the stethoscope. Furthermore, the suspension also provides the necessary degrees of freedom to adapt to the changing anatomy of each patient, enabling the stethoscope head to lie flat across patients. To transmit the signals to the remote station, the real-time computer of stage 1 and the microphone are connected using a UR824 sound interface (Steinberg, Hamburg, Germany).

*Percussion* As there are currently no instruments available for automated or telecontrolled percussion, a designated device had to be designed from scratch. The instrument provides a specially developed mechanism that is based on conventional percussion. The main components of the instrument are an electric drive, a percussion element, a plessimeter, and a heavy-duty microphone. The percussion element, which is pivoted on a rotation spring, is preloaded with the help of the drive. As soon as the percussion element is no longer in engagement with the drive, the percussion element is accelerated by the spring and hits the plessimeter. The microphone, which is mounted next to the plessimeter, records the knocking noises. The impact frequency can be defined via the drive speed and the intervention geometry. As for the auscultation adapter, the microphone is connected to the real-time computer of stage 1 via the sound interface.

*Palpation* As it was already the case for percussion, the absence of a commercial tele-instrument for palpation led to the development of a new telediagnostic palpation-device. The aim of the development was a sensitive adapter that enables the conventional palpation of the patient by the doctor. The implementation resembles the shape of a joystick, modified with large radii to minimize the risk of injury to the patient. The 3-DOF sensor is integrated into the instrument revolver providing the doctor with information about the condition of the patient’s tissue and underlying organs, giving the adapter the necessary sensitivity. The doctor receives feedback about the applied force both via the force feedback of the *Leader* as well as by the control interface of the remote cockpit.

#### Conventional procedures

Stage 3 overlaps spatially with stage 2 and also uses the examination couch. In addition, stage 3 provides a protected exit from the examination cabin to the surrounding area. This enables the medical assistant to take a blood sample from the patient without entering the examination cabin.

*Blood sample* For blood sampling, a take-out handle is provided at the level of the examination couch in one of the transparent walls of the cabin. A rubber glove is attached to the handle, comparable to the structure of a glove box. The glove is secured against slipping out by means of a safety ring in the wall of the examination cubicle. For blood withdrawal, the patient puts his arm into the glove and the medical assistant opens the glove at a designated point. This enables a conventional but contact-minimized blood sampling. The transparency of the pane is unavoidable due to the invasiveness and the trust required by the patient. Robotic solutions for blood sampling were rejected due to the degree of invasiveness and the missing sensitivity regarding robotics.

## Discussion

Results of the survey encouraged us in the necessity of a comprehensive telediagnostic system, especially regarding the infection risks in a pandemic situation. Notably, the current process chain in medical checkups is based on examination methods with a huge discrepancy in objectiveness. For some procedures such as blood pressure measurement or pulse oximetry sophisticated devices, fairly easy integrable in a telemedical setup, are available. For others, such as percussion or auscultation, results rely heavily on the doctors’ individual capabilities and interpretation skills, with no significant technical advances for decades. Thoughtful digitization that goes along with a telemedical transformation can help to standardize examinations and quantify results, opening up further possibilities for assisted or automated analysis. Thus, from a medical perspective, telediagnostic works at least as well as conventional examinations. However, regarding the actual implementation itself, several technical and logistical shortcomings were recognized due to the telemedical setup. Compared to the conventional implementation the assessment of the medical history, otoscopy, throat inspection, palpation, percussion, and auscultation show the shortcomings listed in Table 1. In contrast, blood pressure, temperature, and oxygen saturation measurement, as well as throat sampling, do not show any drawbacks in their current state of development compared to a conventional examination. Even though infectious risks have been reduced to a minimum, the crucial procedure of blood sampling has still to be dealt with manually, impeding a holistic telemedical transformation. Furthermore, the setup requires a thorough cleanup after each patient and therefore relies on on-site staff. For future improvements, non-contact devices such as thermal imaging systems should be considered.

**Table 1:** Shortcomings of the implementation of the telemedical diagnostic framework

Procedure	Shortcomings
*Assisted*	
Assessment of the medical history	Missing sense of smell
Blood pressure measurement	None
Temperature measurement	None
Oxygen saturation measurement	None
Otoscopy	Outcome depends on the patient’s pose
*Teleoperated*	
Throat swap	None
Throat inspection	Outcome depends on the patient’s pose
Auscultation	Interfering noise
	Repeatability in position
	Prolonged duration
Percussion	Interfering noise
	Repeatability in position
	Duration of handling
Palpation	Sensitivity
	Repeatability in position
	Duration of handling
*Conventional*	
Blood sampling	None

The blood pressure, temperature, and oxygen saturation measurement as well as the swab and blood sampling show no technical limitations and could be carried out without any difficulties within the implemented setup. The assessment of the medical history, otoscopy, throat inspection, palpation, percussion, and auscultation, on the other hand, show shortcomings compared to the conventional examination

Using teleoperated robotic systems is a skillful way to deal with the patients’ individual anatomy. Due to the rather high costs of such systems compared to conventional examination procedures, a widespread use is impeded to the present day. Even though the first clinical trials have proven the feasibility of automated swaps, precision has yet to be confirmed by a clinical study.

Within the current status of our work, the hygienic concept is only partially implemented since some aspects require extensive infrastructure that goes beyond our aspired proof of feasibility. Open issues include, inter alia, a sensible strategy for disinfection, and, more importantly, since coronavirus is airborne, a proper ventilation system.

Apart from a system meeting all proposed medical, technical, and hygienic requirements, it remains yet to be seen how patients accept a mere telediagnostic examination. While tele-consultation itself is positively perceived by most patients, especially the direct interaction with a moving robot would be a new experience [9–11]. In surgery, where remotely controlled robots are already used for invasive interventions, studies have proven positive patient acceptance. The systems are often associated with higher precision and a reduced rate of complications. However, conventional surgery was often preferred and results are not directly transferable, as patients are not under anesthesia during a physical examination and have to directly interact [12–14].

Despite the mentioned drawback, we are confident, that our system provides a valuable foundation for future developments in telediagnostics.

## Conclusion

We introduced a proof of concept for the telemedical examination of potentially infectious patients during the COVID-19 pandemic. In the created framework, necessary examinations are divided into three classes: assisted, teleoperated, and conventional procedures. Assisted procedures would be carried out by the patients themselves under the guidance of the physician, whereas teleoperated procedures would be carried out purely by the doctor. Conventional procedures would remain in the hands of medical assistants. For teleoperated examinations, robotic arms would have to be remotely controlled by a doctor. With the help of various robotic instruments, a contactless standardized initial examination could be carried out by the physician.

The search for existing telediagnostic medical devices revealed the actual backwardness of telediagnostics. For the implementation of the telediagnostic Framework, some robotic instruments had to be developed from scratch, while others were modified as available isolated solutions often do not provide interfaces for the integration into an integrative network. Thus, the platform serves as a basis for future developments of telemedical diagnostic tools.

The necessity and demand for telediagnostics have become stronger than ever, particularly in the context of the COVID-19 pandemic. But the reduction in infection rates among clinical staff and patients is only one advantage of contactless examinations. Once such a system works reliably, potential applications range from underprivileged and infrastructural weak regions to areas difficult to reach, such as offshore oil and gas production facilities or cargo ships. The presented framework with its modular approach thereby provides universal principles for future developments in telemedicine, with a foundation ready to be adapted to use-case specific needs. Our major next step will be an in-depth evaluation of the proposed framework and its implementation using multiple medical specialist and voluntary patients.

## Data Availability

The used survey form and individual results can be provided on request.
